# Immunotherapy-related cardiovascular toxicity: from mechanisms to management

**DOI:** 10.3389/fphar.2026.1762011

**Published:** 2026-02-04

**Authors:** Lifeng Xiao, Weitong Liu, Yanchen Ji, Qiufeng Li, Junqing Pan, Renxian Xie

**Affiliations:** 1 Department of Emergency, Cancer Hospital of Shantou University Medical College, Shantou, China; 2 Department of Radiation Oncology, Jieyang People’s Hospital, Jieyang, China; 3 Shantou University Medical College, Shantou, China; 4 Department of Radiation Oncology, Cancer Hospital of Shantou University Medical College, Shantou, China

**Keywords:** cardio-oncology, cardiotoxicity, cytokine release syndrome, heart failure, immune checkpoint inhibitors, immune-related adverse events, myocarditis

## Abstract

Cancer immunotherapy, including immune checkpoint inhibitors and CAR-T cell therapy, has revolutionized oncology but is associated with a broad spectrum of cardiovascular toxicities. This review comprehensively examines the current landscape of these adverse events, which range from myocarditis, pericardial disease, and arrhythmias to heart failure. We delve into the underlying pathophysiological mechanisms, such as T-cell-mediated cross-reactivity via molecular mimicry and cytokine-mediated injury in cytokine release syndrome. The article critically appraises strategies for risk stratification, vigilant monitoring using biomarkers and advanced imaging, and management protocols that encompass immunosuppression, targeted biological therapies, and supportive care. Furthermore, we explore the complex interplay with vaccinations and infections and highlight promising future directions, including novel therapeutic targets, preventive strategies, and advanced monitoring technologies. Ultimately, this review underscores the necessity of a proactive and multidisciplinary cardio-oncology framework to mitigate cardiovascular risks while preserving the anticancer efficacy of immunotherapies.

## 1 Introduction

Immune checkpoint inhibitors (ICIs) have revolutionized cancer treatment by harnessing the immune system to combat malignancies. These therapies, including antibodies targeting programmed cell death protein 1 (PD-1), programmed cell death-ligand 1 (PD-L1), and cytotoxic T-lymphocyte-associated protein 4 (CTLA-4), have demonstrated remarkable efficacy across various tumor types (Liu L. et al., 2024; Panuccio et al., 2024a). However, the enhanced immune activation can lead to immune-related adverse events (irAEs) affecting multiple organ systems, with cardiovascular toxicities representing particularly serious complications (Pi et al., 2024; Keam et al., 2024). Among these, immunotherapy-induced heart failure emerges as a critical concern due to its potential for rapid progression and high mortality rates (Izumi et al., 2024; Nielsen et al., 2024).

The spectrum of cardiovascular complications associated with cancer immunotherapy extends beyond myocarditis to include various forms of cardiac dysfunction that may culminate in heart failure (Munir et al., 2024; Tocchetti et al., 2024). While initial attention focused on ICI-related myocarditis, recent evidence suggests that more subtle forms of cardiac impairment may occur, potentially progressing to overt heart failure (Braghieri et al., 2025; Delombaerde et al., 2024). The complex interplay between enhanced immune activation and cardiovascular homeostasis creates a challenging clinical scenario where early detection and intervention become paramount for optimal patient outcomes (Chen Z. et al., 2024; Toublanc et al., 2024).

This review comprehensively examines the current understanding of immunotherapy-related cardiovascular toxicity, encompassing epidemiological patterns, underlying mechanisms, diagnostic approaches, management strategies, and future directions. By synthesizing evidence from recent clinical studies and mechanistic investigations, we aim to provide clinicians with a practical framework for recognizing, monitoring, and treating this potentially fatal complication while highlighting critical knowledge gaps that warrant further investigation.

## 2 The spectrum of cardiovascular toxicities associated with cancer immunotherapy

Cancer immunotherapy has unveiled a diverse array of cardiovascular complications that extend beyond the initially recognized myocarditis. The cardiovascular toxicities associated with immune checkpoint inhibitors encompass a broad clinical spectrum, ranging from subclinical cardiac biomarker elevations to fulminant heart failure and fatal arrhythmias (Nielsen et al., 2024; Milutinovic et al., 2024). Myocarditis remains the most extensively studied cardiotoxicity, with incidence rates varying from 0.3% to 1.14% in clinical trials but potentially higher in real-world settings (Lindsay et al., 2025; Li et al., 2025). Combination ICI therapy demonstrates significantly increased risk compared to monotherapy, with hazard ratios reaching 2.38 for myocarditis development (Lindsay et al., 2025).

Beyond inflammatory cardiac conditions, ICIs are associated with various forms of cardiac dysfunction that may progress to heart failure. Pericardial diseases, including pericarditis and pericardial effusions, occur with notable frequency (Piazza et al., 2025; Li and Li, 2024). Arrhythmias represent another significant concern, with atrial fibrillation being the most commonly reported cardiac disorder in some analyses (Koecke et al., 2024). Vascular events, including vasculitis and thromboembolic phenomena, further expand the cardiovascular toxicity profile (Grabska et al., 2024).

Chimeric antigen receptor (CAR) T-cell therapy introduces additional cardiovascular considerations, primarily driven by cytokine release syndrome (CRS) (Daryanani et al., 2024). The pooled incidence of cardiovascular events following CAR-T cell therapy includes 54% for arrhythmias, 30% for heart failure, and 20% for cardiomyopathy. These events frequently occur in the context of CRS, with patients experiencing CRS grade ≥2 having a 2.36-fold increased risk of cardiovascular complications (Maleki et al., 2024). The hemodynamic stress from CRS, combined with direct inflammatory effects on cardiac tissue, creates a perfect storm for cardiac dysfunction development.

The timing of cardiovascular event onset varies considerably across immunotherapeutic approaches. ICI-related events typically occur within the first few months of treatment initiation, with median onset around 49 days (Lipe et al., 2024; Eldani et al., 2024). In contrast, CAR-T cell associated cardiovascular complications generally manifest within days to weeks following infusion, closely aligned with CRS development (Daryanani et al., 2024). This temporal pattern has important implications for monitoring strategies and intervention timing.

Understanding the full spectrum of immunotherapy-associated cardiovascular toxicities is essential for developing comprehensive screening and management protocols. The spectrum and key characteristics of these toxicities are summarized in Table 1. The heterogeneity in clinical presentation, combined with the potential for rapid deterioration, necessitates heightened awareness among oncologists and cardiologists alike to ensure prompt recognition and treatment of these potentially devastating complications.

**TABLE 1:** Spectrum and characteristics of cardiovascular toxicities associated with cancer immunotherapy.

Toxicity type	Associated therapy	Key clinical features
Myocarditis (Lindsay et al., 2025; Li et al., 2025)	ICIs (especially combination therapy)	Chest pain, dyspnea, troponin elevation, LV dysfunction
Pericardial Disease (Piazza et al., 2025; Li and Li, 2024)	ICIs	Pericarditis, pericardial effusion, tamponade
Arrhythmias (Koecke et al., 2024; Maleki et al., 2024)	ICIs, CAR-T	Atrial fibrillation, ventricular arrhythmias
Heart Failure/Cardiomyopathy (Izumi et al., 2024; Tocchetti et al., 2024; Maleki et al., 2024)	ICIs, CAR-T	LVEF reduction, edema, biomarker elevation
Vascular Events (Grabska et al., 2024)	ICIs	Vasculitis, thromboembolism
CRS-related Cardiac Dysfunction (Munir et al., 2024; Daryanani et al., 2024; Maleki et al., 2024)	CAR-T	Hypotension, capillary leak, cytokine-driven myocardial suppression

Abbreviations: ICI, immune checkpoint inhibitor; CAR-T, Chimeric Antigen Receptor T-cell; CRS, cytokine release syndrome; LVEF, left ventricular ejection fraction.

## 3 Pathophysiological mechanisms underlying immunotherapy-induced cardiotoxicity

The pathophysiological mechanisms driving immunotherapy-induced cardiotoxicity involve complex interactions between immune activation and cardiovascular homeostasis. The fundamental principle underlying these toxicities revolves around the disruption of immune checkpoint pathways that normally maintain self-tolerance and prevent autoimmunity (Gergely et al., 2024). By inhibiting CTLA-4, PD-1, or PD-L1, ICIs remove inhibitory signals that constrain T-cell activation, potentially unleashing autoreactive T-cells that recognize cardiac antigens (Blum et al., 2024).

Molecular mimicry represents a proposed mechanism whereby T-cells primed against tumor antigens cross-react with similar epitopes expressed on cardiac tissue (Gergely et al., 2024). This phenomenon may explain why myocarditis frequently coincides with other irAEs, particularly myositis and myasthenia gravis, in what is termed the MMM overlap syndrome. The shared epitopes between cardiac muscle, skeletal muscle, and neuromuscular junction components create opportunities for cross-reactivity that manifests as concurrent multi-organ inflammation (Lipe et al., 2024; Beecher et al., 2024).

Cytokine-mediated injury constitutes another significant pathway in immunotherapy-related cardiotoxicity. The analysis of heart tissue from patients with ICI-myocarditis reveals increased frequencies and co-localization of cytotoxic T-cells, conventional dendritic cells, and inflammatory fibroblasts. Simultaneously, peripheral blood shows decreased frequencies of plasmacytoid dendritic cells and B-lineage cells but increased mononuclear phagocytes (Blum et al., 2024). This altered immune cell distribution creates a pro-inflammatory milieu that can compromise cardiac function.

The role of specific cytokine pathways continues to be elucidated. Evidence suggests involvement of type I and type III immune signatures, with particular emphasis on interleukin (IL)-17A expressing CD4 T-cells (Dimitriou et al., 2024). In patients experiencing irAEs, proteomic analyses demonstrate aberrant T-cell activity with differential expression of these immune signatures. The finding that anti-IL-17A administration resolved adverse events in two patients with mild myocarditis, colitis, and skin rash provides preliminary evidence for targeting specific cytokine pathways.

Beyond inflammatory mechanisms, direct cardiomyocyte injury contributes to cardiac dysfunction. Histological examination of myocarditis cases reveals varying degrees of cardiomyocyte injury, ranging from pronounced to absent, along with different types of inflammatory infiltrates (Omori et al., 2024). Those with cardiomyocyte injury present clinically with older age, balanced sex distribution, less frequent chest pain, and lower left ventricular ejection fraction, and more frequently develop fulminant myocarditis requiring mechanical circulatory support.

Emerging evidence also suggests a potential interplay between immune checkpoint inhibition and the progression of coronary atherosclerosis. The systemic inflammatory state induced by ICIs, characterized by elevated cytokines and activated T-cells, may exacerbate endothelial dysfunction, promote plaque instability, and accelerate atherosclerotic progression (Baitsch et al., 2011; Suero-Abreu et al., 2022). This interaction raises the possibility that ICI-related cardiotoxicity may not only manifest as acute myocarditis but also as a worsening of underlying coronary artery disease, potentially leading to acute coronary syndromes (Thuny et al., 2022). In this context, timely coronary revascularization may play a crucial role in managing significant coronary lesions exacerbated by immunotherapy, aiming to restore blood flow, stabilize vulnerable plaques, and prevent ischemic complications. Recent meta-analyses underscore the importance of revascularization in improving cardiovascular outcomes in patients with chronic coronary syndromes, highlighting its relevance even in the setting of cancer therapy-induced vascular injury (Panuccio et al., 2024b).

The cardiovascular effects of cytokine release syndrome in CAR-T cell therapy involve additional mechanisms. CRS generates massive inflammatory responses with elevated levels of IL-6, interferon-γ, and other cytokines that can directly suppress myocardial function and promote vascular leakage (Munir et al., 2024; Camilli et al., 2024). This cytokine storm can induce cardiac dysfunction through both direct myocardial depression and indirect effects on vascular integrity and afterload (Maleki et al., 2024). The key pathophysiological mechanisms underlying immunotherapy-induced cardiotoxicity are outlined in Table 2.

**TABLE 2:** Key pathophysiological mechanisms of immunotherapy-induced cardiotoxicity.

Mechanism	Key components/Pathways	Evidence/Manifestations
T-cell-mediated cross-reactivity (Molecular mimicry) (Lipe et al., 2024; Gergely et al., 2024; Beecher et al., 2024)	Shared epitopes between tumor and cardiac antigens; MMM overlap syndrome (myocarditis, myositis, myasthenia gravis)	Co-localization of cytotoxic T-cells in heart tissue; concurrent multi-organ inflammation
Cytokine-mediated injury (Blum et al., 2024; Dimitriou et al., 2024)	IL-6, IFN-γ, IL-17A, TNF-α; altered immune cell distribution (↑mononuclear phagocytes, ↓plasmacytoid DCs)	CRS in CAR-T; cytokine storm leading to myocardial depression and vascular leakage
Direct cardiomyocyte injury (Omori et al., 2024)	Varying degrees of cardiomyocyte necrosis/apoptosis; inflammatory infiltrates	Histological findings; older age, lower LVEF, fulminant course
Altered immune checkpoint signaling (Gergely et al., 2024; Blum et al., 2024)	Inhibition of CTLA-4, PD-1, PD-L1 unleashes autoreactive T-cells	Foundational mechanism for ICI-related toxicity
Cytokine profiling in refractory cases (Tan et al., 2024)	IL-6 decrease with TNF-α increase may guide therapy (infliximab)	Serum cytokine monitoring can inform targeted intervention

Abbreviations: CTLA-4, Cytotoxic T-Lymphocyte-Associated protein 4; PD-1, Programmed cell Death protein 1; PD-L1, Programmed Death-Ligand 1; MMM, myocarditis, Myositis, Myasthenia gravis; IL-6, Interleukin-6; IFN-γ, Interferon-gamma; IL-17A, Interleukin-17A; TNF-α, Tumor Necrosis Factor-alpha; DCs, Dendritic Cells; LVEF, left ventricular ejection fraction.

Understanding these multifaceted mechanisms is crucial for developing targeted therapeutic approaches that mitigate cardiotoxicity without compromising antitumor efficacy. The complexity of these pathways underscores the need for continued mechanistic research to identify optimal intervention points for preventing and treating immunotherapy-related heart failure.

## 4 Risk stratification, monitoring, and diagnostic approaches

Effective management of immunotherapy-related cardiovascular toxicity necessitates robust risk stratification, vigilant monitoring, and accurate diagnostic approaches. Current evidence suggests several potential biomarkers and clinical factors that may help identify patients at elevated risk before and during treatment (Delombaerde et al., 2024). Baseline cardiovascular risk factors, including hypertension and dyslipidemia, appear to increase susceptibility to cardiac events during immunotherapy (Salas et al., 2024; Peng et al., 2024). Older age, male sex, and prior radiation therapy have also been associated with increased major adverse cardiovascular event risk (Li et al., 2025).

Multimodal biomarker assessment shows promise for risk prediction. A scoring strategy incorporating four biomarkers—N-terminal-pro-B-type-natriuretic-peptide (NT-proBNP) (≥125 pg/mL), cardiac troponin T (≥6 ng/L), high-sensitivity C-reactive protein (≥3 mg/L), and coronary artery calcium score (>10 U)—demonstrated ability to stratify patients according to ICIs-related cardiotoxicity risk. Patients with very high scores (4 points) showed 7.29 to 8.83-fold higher risk compared to those with low scores (Chen Z. et al., 2024). An overview of risk stratification tools and monitoring approaches is provided in Table 3. These biomarkers also predicted earlier onset and higher incidence of cardiotoxicity, suggesting potential utility in pre-treatment assessment.

**TABLE 3:** Risk stratification and monitoring tools for immunotherapy-related cardiovascular toxicity.

Category	Parameters/Biomarkers	Utility/Notes
Baseline Risk Factors (Li et al., 2025; Salas et al., 2024; Peng et al., 2024)	Age (>65 years), male sex, prior radiation therapy, hypertension, dyslipidemia	Associated with increased MACE risk; inform pre-treatment assessment
Biomarker Panel for Risk Scoring (Peng et al., 2024)	NT-proBNP (≥125 pg/mL), cTnT (≥6 ng/L), hs-CRP (≥3 mg/L), CAC score (>10 U)	4-point scoring system; high score (Keam et al., 2024) → 7.29–8.83× increased risk
Monitoring during Therapy (Tomsitz et al., 2025)	High-sensitivity troponin T (hs-TnT) before each infusion	Screening detected irMyocarditis in 4.1%–9.5% of patients; may reduce mortality
Imaging Modalities (Celebi et al., 2024; Pozzessere et al., 2024)	Echocardiography (LVEF, GLS), Cardiac MRI (tissue characterization)	Cornerstone for function and structure; CMR offers high diagnostic precision
Electrocardiographic Monitoring (Qin et al., 2024)	ECG abnormalities (arrhythmias, conduction delays)	Often present in cardiotoxicity; combined with troponin predicts MACE
Novel Surveillance Tools (Deady et al., 2024)	Computable phenotype algorithms (electronic health record analysis)	PPV ∼58.3% for myocarditis/pericarditis detection; enables real-time monitoring

Abbreviations: MACE, major adverse cardiovascular event; NT-proBNP, N-terminal pro-B-type Natriuretic Peptide; cTnT, cardiac Troponin T; hs-CRP, high-sensitivity C-Reactive Protein; CAC, coronary artery calcium; hs-TnT, high-sensitivity Troponin T; LVEF, left ventricular ejection fraction; GLS, global longitudinal strain; CMR, cardiac magnetic resonance; ECG, electrocardiogram; PPV, positive predictive value.

Monitoring during therapy primarily relies on cardiac biomarkers and imaging modalities. Troponin surveillance has emerged as a valuable tool for early detection of myocardial injury. Prospective screening with high-sensitivity cardiac troponin T (hs-TnT) before each ICI infusion identified irMyocarditis in 4.1% of patients with normal baseline hs-TnT and 9.5% of those with elevated baseline. Importantly, no fatalities occurred among the 16 irMyocarditis patients detected through this screening approach, suggesting potential mortality reduction through early intervention (Tomsitz et al., 2025).

Echocardiography remains a cornerstone for monitoring cardiac function during immunotherapy. Left ventricular ejection fraction (LVEF) and global longitudinal strain (GLS) represent standard parameters for assessment, though their sensitivity for early detection requires further validation. In one study, reduced left ventricular GLS did not demonstrate significant predictive value for ICI-related myocarditis or cardiac dysfunction, though the small sample size limited definitive conclusions (Celebi et al., 2024). Advanced imaging modalities, particularly cardiac magnetic resonance (CMR) with tissue characterization, provide additional diagnostic precision for detecting inflammatory cardiac conditions (Pozzessere et al., 2024; Efentakis et al., 2024).

Electrocardiographic monitoring offers another valuable tool, with abnormalities frequently present in patients developing cardiotoxicity (Qin et al., 2024). In patients with ICI-related myocarditis, electrocardiogram changes combined with elevated troponin levels helped predict major adverse cardiac events. The combination of these readily available parameters may facilitate early intervention before overt heart failure develops.

For CAR-T cell recipients, monitoring strategies must account for the different timing and mechanism of cardiotoxicity. The strong association between CRS and cardiovascular events suggests that close monitoring should be instituted during and after CRS development (Daryanani et al., 2024). Cardiac biomarkers, including natriuretic peptides and troponins, along with echocardiographic assessment, appear valuable for detecting emerging cardiac dysfunction in this context (Camilli et al., 2024).

The emergence of novel monitoring technologies, including computable phenotype algorithms for automated adverse event detection, may enhance surveillance capabilities (Deady et al., 2024). Such approaches demonstrated positive predictive values around 58.3% for myocarditis/pericarditis detection, though performance varied across healthcare systems. These tools could potentially be integrated into electronic health records to facilitate real-time monitoring of large patient populations.

Despite these advances, significant knowledge gaps remain in optimal monitoring strategies. The frequency of assessments, the most cost-effective approach for different risk groups, and the integration of novel biomarkers require further investigation. Standardized protocols for surveillance and diagnosis will be essential for improving outcomes of patients experiencing immunotherapy-related cardiovascular toxicity.

## 5 Management strategies for immunotherapy-related cardiovascular adverse events

The management of immunotherapy-related cardiovascular adverse events requires a multidisciplinary approach involving oncologists, cardiologists, and other specialists. The cornerstone of management involves immediate discontinuation of the offending agent and initiation of immunosuppressive therapy, typically starting with high-dose corticosteroids (Tan et al., 2024; Liu Q. et al., 2024). For ICI-associated myocarditis, prompt initiation of methylprednisolone (1–2 mg/kg) is recommended, with escalation to other immunosuppressive agents for severe or refractory cases (Alghabba et al., 2025).

The management approach must be tailored according to severity grading. For grade 2 or higher cardiotoxicity, current guidelines recommend holding ICI therapy and initiating prednisone at 1–2 mg/kg/day. For severe (grade 3–4) events, hospitalization and intravenous methylprednisolone pulse therapy (1,000 mg daily for 3–5 days) is indicated. Refractory cases may require additional immunosuppressive agents, including mycophenolate mofetil, infliximab, or abatacept (Nielsen et al., 2024). A summary of management strategies based on severity grading is presented in Table 4.

**TABLE 4:** Management strategies based on severity of immunotherapy-related cardiovascular adverse events.

Severity grade	Clinical features	Recommended management
Grade 1	Asymptomatic biomarker elevation	Continue ICI with close monitoring
Grade 2 (Nielsen et al., 2024; Alghabba et al., 2025)	Mild symptoms; biomarker elevation	Hold ICI; start prednisone 1–2 mg/kg/day
Grade 3–4 (Nielsen et al., 2024; Alghabba et al., 2025)	Severe symptoms, hemodynamic compromise, life-threatening	Hospitalize; IV methylprednisolone 1 g/day for 3–5 days; consider additional immunosuppressants (mycophenolate, infliximab, abatacept)
Refractory Cases (Tan et al., 2024)	Poor response to steroids	Target cytokine profiles (infliximab if TNF-α elevated); consider tofacitinib (JAK-STAT inhibition)
CAR-T associated (CRS-driven) (Munir et al., 2024; Liu et al., 2024b)	Hypotension, cytokine storm	First-line: tocilizumab (anti-IL-6R); supportive care; manage cardiac dysfunction similarly to ICI cases
Supportive Care for Heart Failure	LV dysfunction, volume overload	Guideline-directed medical therapy (beta-blockers, ACEi/ARB, diuretics); mechanical circulatory support if needed
Rechallenge Considerations (Eldani et al., 2024)	After complete resolution of toxicity	Possible in selected patients under close monitoring; recurrence risk exists

Abbreviations: ICI, immune checkpoint inhibitor; IV, intravenous; LV, left ventricle; CRS, cytokine release syndrome; IL-6R, Interleukin-6, receptor; ACEi, Angiotensin-Converting Enzyme inhibitor; ARB, angiotensin receptor blocker.

Emerging evidence supports targeted approaches based on specific cytokine profiles. In steroid-refractory myocarditis cases where serum interleukin (IL)-6 decreased by >50% while tumor necrosis factor-α (TNF-α) increased, infliximab administration resulted in gradual alleviation of myocarditis. This suggests that cytokine monitoring might guide selection of specific cytokine inhibitors for refractory cases (Tan et al., 2024).

For cardiovascular complications associated with CAR-T cell therapy, management focuses on controlling the underlying CRS. Tocilizumab, an IL-6 receptor antagonist, serves as first-line therapy for moderate to severe CRS (7). The management of subsequent cardiac dysfunction follows similar principles to ICI-related events, though the context of profound cytokine release may necessitate additional supportive measures.

Supportive care represents a critical component of management, particularly for patients developing heart failure. Standard heart failure therapies, including beta-blockers, angiotensin-converting enzyme inhibitors, and diuretics, should be implemented according to current guidelines (Tocchetti et al., 2024). In severe cases with hemodynamic compromise, mechanical circulatory support may be necessary as a bridge to recovery (Izumi et al., 2024).

The question of rechallenge after cardiovascular toxicity remains complex and inadequately studied. Limited evidence suggests that rechallenge may be possible in selected patients after complete resolution of the adverse event and under close monitoring. In one series, among 15 patients retreated with ICIs after severe irAEs (including cardiovascular and neurological events), 73% remained free of subsequent irAEs, while 13% experienced recurrence of the initial irAE and 13% developed new irAEs (Eldani et al., 2024). However, these decisions must be individualized, weighing the oncological benefits against potential risks.

Novel preventive strategies are emerging from mechanistic studies. Statins, particularly atorvastatin, demonstrated protective effects in preclinical models of ICI-related cardiotoxicity (Efentakis et al., 2024; Jiang et al., 2024). The pleiotropic effects of statins, including antioxidative, anti-inflammatory, antifibrotic, endothelial protective, and immune modulatory properties, position them as promising candidates for cardioprotection during immunotherapy. Similarly, glucagon-like peptide-1 agonists showed reduced major adverse cardiovascular events in cancer patients with type 2 diabetes receiving ICIs (Chiang et al., 2025).

The management of immunotherapy-related cardiovascular events continues to evolve as our understanding of mechanisms expands. While corticosteroids remain foundational, targeted approaches based on specific pathological processes hold promise for improving outcomes while preserving anticancer efficacy. Multidisciplinary collaboration is essential for optimizing management strategies for these complex patients.

## 6 The interplay between vaccinations, infections, and immunotherapy cardiotoxicity

The relationship between vaccinations, infections, and immunotherapy-related cardiotoxicity presents a complex clinical scenario with significant implications for patient management. Emerging evidence suggests that viral infections, particularly SARS-CoV-2, may influence the development and severity of immune-related adverse events (Sumi et al., 2024; Gradone et al., 2024; Watson et al., 2024). Similarly, vaccinations—especially COVID-19 vaccines—have been associated with rare cardiovascular complications that may intersect with immunotherapy toxicities (Omori et al., 2024; Weirauch et al., 2025).

The temporal relationship between COVID-19 vaccination and ICI toxicity warrants careful consideration. Cases of severe myositis with myocarditis have been reported within 1 month of COVID-19 mRNA vaccination followed by PD-1 inhibition (Watson et al., 2024). Analysis of T-cell receptor repertoires from these patients revealed a high degree of clonal expansion and public clones between cases, with several T-cell clones expanded within skeletal muscle putatively recognizing viral epitopes. All patients had recently received COVID-19 mRNA booster vaccination prior to treatment and were positive for SARS-CoV-2 Spike antibody, suggesting potential interaction between vaccine-induced immune activation and ICI effects.

The incidence of vaccine-associated myocarditis/pericarditis must be contextualized within the broader benefit-risk profile of vaccination. The reporting rate for myocarditis/pericarditis following original monovalent COVID-19 mRNA vaccination was approximately 6.91 per million doses, while the rate for bivalent vaccination was significantly lower at 1.24 per million doses (Chen C. et al., 2024). These risks must be balanced against the established cardiovascular benefits of vaccination against infectious diseases.

Influenza vaccination demonstrates particular relevance for cancer patients receiving immunotherapy. Vaccination reduces the risk of major adverse cardiovascular events in patients with ischemic heart disease (Liu et al., 2025). For heart failure patients, influenza vaccination represents a cost-effective intervention that reduces hospitalizations and mortality (Miró et al., 2025; Zhao et al., 2024). In immunocompromised cancer patients, influenza vaccination was associated with reduced overall mortality across all subgroups, with the most pronounced reduction in patients with hematological cancer aged <65 years (adjusted hazard ratio 0.66) (Amdisen et al., 2025).

The potential interaction between infections and ICI toxicity extends beyond viral pathogens. Co-occurring infections in cancer patients treated with checkpoint inhibitors significantly increase the risk of immune-related adverse events. Analysis of over nineteen million reports revealed statistically significant associations between irAEs and concurrent infectious diseases for five out of seven ICIs treatments (Grabska et al., 2024). This finding suggests that infections may lower the threshold for developing irAEs, possibly through enhanced immune activation or molecular mimicry mechanisms.

The management of patients receiving immunotherapy during periods of prevalent infections requires careful consideration. The benefits of vaccination generally outweigh potential risks, but timing relative to immunotherapy cycles may influence both efficacy and safety (Tsang et al., 2024; Le Vu et al., 2024). For COVID-19 vaccination, extending the dosing interval has been employed to reduce myocarditis risk in adolescents, though this approach must balance protection against infection during the extended intra-dose period.

Understanding these complex interactions is essential for optimizing patient care during immunotherapy. Vaccination remains a critical component of supportive care for cancer patients, but awareness of potential intersections with immunotherapy toxicity can guide appropriate monitoring and management strategies.

## 7 Novel therapeutic targets and future directions in cardio-oncology

The rapidly evolving field of cardio-oncology continues to identify novel therapeutic targets and future directions for managing immunotherapy-related cardiovascular toxicity. Advances in understanding the molecular mechanisms underlying these adverse events have revealed several promising approaches for prevention and treatment (Kong et al., 2024). The recognition that specific immune pathways drive cardiotoxicity provides opportunities for targeted interventions that may preserve antitumor efficacy while mitigating cardiovascular risks.

The IL-17A pathway represents a compelling target based on evidence from patients experiencing irAEs (Dimitriou et al., 2024). The finding that anti-IL-17A administration resolved adverse events in two patients with mild myocarditis, colitis, and skin rash provides proof-of-principle for this approach. Further investigation in clinical trials is warranted to establish the efficacy and safety of IL-17 inhibition for immunotherapy-related cardiotoxicity. Similarly, the JAK-STAT pathway has emerged as another potential target, with tofacitinib showing promising clinical efficacy in patients experiencing irAEs, particularly those who failed to respond to steroids or experienced relapse during steroid tapering (Liu Q. et al., 2024).

Bioorthogonal metabolic engineering-driven extracellular vesicle redirecting (Biomeder) represents an innovative approach for reversing ICI-associated cardiotoxicity (Fan et al., 2024). This strategy involves *in situ* engineering of tumor-derived extracellular vesicles with myocardial-targeting peptides, which accumulate in the heart and reverse the immune environment by increasing PD-L1 levels in cardiomyocytes and/or directly inhibiting T-cell activity. Remarkably, this approach not only alleviated cardiotoxicity but also enhanced antitumor efficacy by disrupting immunosuppression in tumors. The technique was successfully expanded to prevent ICI-induced type 1 diabetes, suggesting potential applicability across various irAEs.

Statins continue to show promise as preventive agents based on their pleiotropic effects. Preclinical studies demonstrated that atorvastatin mitigated functional deficits in models of ICI-induced cardiotoxicity by inhibiting endothelial dysfunction (Efentakis et al., 2024). The antioxidative stress, anti-inflammatory, antifibrotic, endothelial protective, and immune modulatory properties of statins position them as attractive candidates for cardioprotection during immunotherapy (Jiang et al., 2024). Prospective clinical trials are needed to validate these findings and establish optimal dosing regimens.

The development of novel immunotherapeutic agents with improved safety profiles represents another important direction. PROTAC-based HPK1 degraders have shown high potency and selectivity for cancer immunotherapy with a low risk of cardiotoxicity and wide safety window in preclinical models (Zhang et al., 2024). Similarly, the CD80 variant immunoglobulin domain Fc fusion protein davoceticept (ALPN-202) was designed to mediate PD-L1-dependent CD28 costimulation while inhibiting PD-L1 and CTLA-4 checkpoints, potentially minimizing systemic T-cell activation and untoward toxicities (Cavalcante et al., 2024). However, cases of fatal myocarditis were reported with this agent in combination with pembrolizumab, highlighting the continued challenges in balancing efficacy and safety.

Advanced monitoring technologies offer additional opportunities for improving patient outcomes. Computable phenotype algorithms enable automated detection of adverse events through analysis of electronic health records, facilitating real-time surveillance (Deady et al., 2024). The implementation of such systems required 200–250 h but achieved positive predictive values of 58.3% for myocarditis/pericarditis detection. Further refinement and validation of these tools could significantly enhance early detection and management of cardiovascular toxicities.

The future of cardio-oncology will likely involve increasingly personalized approaches based on individual risk profiles, tumor characteristics, and specific immunotherapeutic regimens. Biomarker-guided therapy, leveraging advances in proteomics, transcriptomics, and immunosequencing, may enable precise targeting of pathological processes while preserving antitumor immunity. Multicenter collaborations and standardized registries will be essential for advancing our understanding of these complex interactions and developing evidence-based management strategies.

## 8 Discussion

The expanding use of cancer immunotherapies has unmasked a significant spectrum of cardiovascular toxicities that demand vigilant clinical attention and a deepened mechanistic understanding. This review synthesizes current evidence indicating that these adverse events, ranging from fulminant myocarditis to chronic cardiomyopathy, are not merely idiosyncratic reactions but are often rooted in dysregulated immune activation. The pathophysiology involves a complex interplay of T-cell-mediated cross-reactivity, cytokine-driven myocardial injury, and direct cardiomyocyte damage, as outlined in the preceding sections. Importantly, the risk extends beyond the widely recognized immune checkpoint inhibitors to include cellular therapies like CAR-T cells, where cytokine release syndrome serves as a potent driver of cardiac dysfunction.

A key clinical imperative that emerges is the necessity for proactive, multidisciplinary collaboration between oncology and cardiology—the core of the cardio-oncology framework. The management algorithms discussed herein, which hinge on early detection via biomarker surveillance and advanced imaging, followed by graded immunosuppression, underscore a paradigm shift from reactive to pre-emptive care. However, significant knowledge gaps persist. The optimal frequency of monitoring, the definitive role of novel biomarkers, and standardized protocols for re-challenging patients after a cardiotoxic event remain areas of active investigation and debate.

Furthermore, the intersecting influences of concurrent infections and vaccinations on immunotherapy toxicity profiles introduce additional layers of complexity to patient management. While vaccinations remain crucial for patient protection, their timing and potential interaction with immune checkpoint pathways warrant careful consideration.

In conclusion, the cardiovascular toxicities associated with cancer immunotherapy are serious but potentially manageable complications. Their successful mitigation hinges on a dual approach: first, the continued elucidation of underlying biological mechanisms to inform targeted therapies; and second, the rigorous implementation of structured surveillance and management protocols in clinical practice. Future research must focus on validating predictive risk models, refining therapeutic strategies to preserve anticancer efficacy, and fostering robust cardio-oncology partnerships to improve overall patient outcomes.

## 9 Future perspectives

Building upon the current understanding and management strategies discussed above, the field of cardio-oncology must now address several critical knowledge gaps and translate mechanistic insights into improved patient outcomes. The immediate future should be directed toward validating targeted therapeutic approaches in prospective clinical trials, particularly for steroid-refractory cases. Agents inhibiting specific cytokine pathways, such as IL-17A or JAK-STAT inhibitors, represent promising candidates based on early mechanistic studies and require rigorous evaluation of their efficacy and safety profiles.

Concurrently, advancing preventive strategies is paramount. Large-scale studies are needed to determine whether prophylactic administration of cardioprotective agents, such as statins, can mitigate toxicity without interfering with anticancer immunity. Parallel efforts must focus on refining risk stratification by discovering and validating predictive biomarkers that integrate genomic, proteomic, and clinical data to identify high-risk patients before initiating immunotherapy.

The integration of advanced monitoring technologies into routine clinical practice presents another crucial frontier. The development and standardization of computable phenotype algorithms for automated toxicity detection within electronic health records could enable real-time surveillance across large populations, facilitating earlier intervention. Furthermore, prospective studies must establish the optimal frequency and modality of cardiovascular monitoring tailored to different immunotherapies and patient risk profiles.

Ultimately, the evolution of cardio-oncology hinges on a deeply personalized, patient-centered approach. This will require not only biomarker-guided therapy but also a nuanced understanding of how tumor biology, host immunogenetics, and cardiovascular comorbidities interact to influence toxicity risk. Strengthening multidisciplinary collaboration through structured cardio-oncology programs and international registries will be essential to generate robust real-world evidence, standardize management protocols, and design the next-generation of clinical trials aimed at preserving the revolutionary benefits of cancer immunotherapy while ensuring cardiovascular safety.
